# Quantifying the Nonclassicality of the Kirkwood–Dirac Quasiprobability Distribution Under Discrete-Time Dynamics

**DOI:** 10.3390/e28040395

**Published:** 2026-04-01

**Authors:** Ziheng Ding, Si-Qi Zhou

**Affiliations:** 1 School of Mathematical Sciences, MOE-LSC, Shanghai Jiao Tong University, Shanghai 200240, China; 2 School of Computer Science and Engineering, Sun Yat-sen University, Guangzhou 510006, China

**Keywords:** temporal KD quasiprobability distribution, nonclassicality measure, spatiotemporal compatibility

## Abstract

The Kirkwood–Dirac (KD) quasiprobability distribution describes any quantum state with respect to the eigenbases of two incompatible observables. While the KD quasiprobability distribution behaves similarly to a classical probability distribution, it can take on negative or nonreal values. Recently, the framework of the *temporal Kirkwood–Dirac quasiprobability distribution* has been proposed, generalizing the KD quasiprobability distribution to arbitrary multi-time quantum processes. In this work, we specifically focus on the temporal KD quasiprobability distribution within the context of two-time dynamics. We begin by constructing a nonclassicality measure derived from the real and imaginary parts of the temporal KD quasiprobability distribution. Next, we establish two uncertainty relations closely linked to this nonclassicality measure, one of which shows that the nonclassicality measure is bounded below by the measurement disturbance caused by the first measurement. Finally, we elucidate the relationships among temporal KD nonclassicality, the spatiotemporal Born rule, and spatiotemporal compatibility.

## 1. Introduction

In classical mechanics, a joint probability distribution effectively characterizes a system in terms of two observables, such as position and momentum. However, in the realm of quantum mechanics, it is impossible to establish a joint probability distribution for two incompatible observables. To address this limitation, quasiprobability distributions, which may assume negative or nonreal values, have emerged as viable alternatives. Notably, the Wigner function in continuous-variable systems has been instrumental in the analysis of quantum states of light [1,2]. In particular, the presence of Wigner negativity has been shown to reveal fundamental connections in quantum information [3,4,5,6,7].

The Kirkwood–Dirac (KD) quasiprobability distribution [8,9] has recently demonstrated remarkable benefits in systems with discrete variables. In the realm of quantum metrology, the negativity inherent in the KD quasiprobability distribution serves to enhance the Fisher information in postselected metrology [10,11]. Within the context of quantum chaos, a generalized KD quasiprobability distribution can indicate the process of scrambling, characterized by the dissemination of information regarding a local perturbation through many-body entanglement [12]. Furthermore, the average work output in a quantum engine exhibits a nearly linear relationship with the imaginary components of the KD quasiprobability distribution [13]. A state is classified as KD positive if its KD quasiprobability distribution exclusively yields non-negative values. A fundamental challenge lies in the characterizations of KD positive states, for which several sufficient or necessary conditions have been identified [14,15,16,17]. The collection of all KD positive states corresponds to the convex hull formed by two specific orthonormal bases under reasonable hypotheses [17]. The KD quasiprobability distribution has also been extended to accommodate more than two orthonormal bases or positive-operator-valued measures (POVMs) [11,12,18].

The framework of “two-time expectation values” was first introduced through the pseudo-density matrix (PDM) formalism in ref. [19]. Later on, various spatiotemporal frameworks have been explored in ref. [20]. Ref. [21] provided a closed-form expression of the PDM based on the coarse-grained measurement scheme for multi-qubit systems across multiple times. This closed-form PDM under coarse-grained measurement is also known as the canonical quantum state over time. Finally, ref. [22] generalized the measurement scheme proposed in ref. [21] to arbitrary finite-dimensional quantum systems. This two-time expectation value also incorporates the evolution of the state through certain channels. Motivated by the observation that the negativity of standard KD distribution is typically associated with the quantum spatial correlations, i.e., correlations encoded in quantum states. We consider how to describe the evolution of the KD quasiprobability distribution across different times and quantum systems. In other words, is there a novel KD quasiprobability distribution that can effectively encapsulate the quantum temporal correlations encoded in a PDM?

Recently, the framework of temporal KD quasiprobability distribution has been proposed in ref. [23], which not only accounts for the varying times at which measurements are performed but also incorporates transitions between different quantum systems. The concept of temporal KD quasiprobability distribution provides a new perspective for addressing KD quasiprobability distributions within a unified spatiotemporal framework, thereby extending the scope of the generalized KD quasiprobability distributions discussed in refs. [11,12,18,24,25]. However, even the KD quasiprobability distribution under two-time dynamics has not been thoroughly explored, particularly regarding its characterization of nonclassicality and its relationships with spatiotemporal compatibility. In this work, we systematically study the temporal KD quasiprobability distribution under two-time dynamics, examining aspects such as nonclassicality, relationships with uncertainty, and causal correlations. Specifically, we establish a measure to quantify the nonclassicality of the temporal KD quasiprobability distribution under two-time dynamics. This measure of nonclassicality is derived from the negativity and imaginary parts of the temporal KD quasiprobability distribution, demonstrating several advantageous properties. Subsequently, it is shown that this nonclassicality measure acts as a lower bound for the Schrödinger uncertainty relation under appropriate selections of observables and states. The nonclassicality measure is also lower-bounded by the measuremnt disturbance caused by the first measurement. We further unveil the close connections among the temporal KD quasiprobability distribution, spatiotemporal Born rule, and spatiotemporal compatibility.

Throughout this work, *A* and *B* are denoted as finite-dimensional quantum systems with Hilbert spaces $H_{A}$ and $H_{B}$, where $\dim\;H_{A}=\dim\;H_{B}=n$. The linear operators acting on these Hilbert spaces are assumed to form $C^{*}$-algebras, which we refer to as A and B. The sets of density operators associated with $H_{A}$ and $H_{B}$ are denoted as $S(H_{A})$ and $S(H_{B})$, respectively. A linear map E:A→B that is completely positive and trace preserving is identified as a quantum channel, with the collection of all quantum channels denoted by CPTP(A,B). The Hilbert–Schmidt adjoint $E^{*}:B\to A$ of the linear map E:A→B is the unique linear map satisfying $\mathrm{Tr}[E{\left(C\right)}^{†}D]=\mathrm{Tr}[C^{†}E^{*}\left(D\right)]$ for all C∈A and D∈B, where the symbol † denotes the conjugate transpose. The set $\mathrm{CPTP}\left(A,B\right)\times S\left(H_{A}\right)$ is denoted by P(A,B), and an element (E,ρ)∈P(A,B) is referred to as a process. A collection of projections $P=\{P_{i}\}$ is said to be a projective measurement if $P_{i}P_{j}=\delta_{ij}P_{i}$ and $\sum_{i}P_{i}=1$, where $\delta_{ij}$ is the Kronecker symbol.

## 2. Temporal KD Quasiprobability Distribution Under Two-Time Dynamics

Before turning to the definition of the temporal KD quasiprobability distribution, it is pertinent to revisit the standard KD quasiprobability distribution established by Kirkwood [8] and Dirac [9]. Let us consider a quantum system characterized by the *n*-dimensional Hilbert space H, which has two orthonormal bases denoted as ${\left\{|a_{i}\rangle \right\}}_{i=1}^{n}$ and ${\left\{|b_{j}\rangle \right\}}_{j=1}^{n}$. The standard KD quasiprobability distribution associated with the bases ${\left\{|a_{i}\rangle \right\}}_{i=1}^{n}$ and ${\left\{|b_{j}\rangle \right\}}_{j=1}^{n}$ for the state ρ∈S(H) is defined as [8,9]:

(1) $$K_{i,j}\left(\rho \right)=\langle b_{j}|a_{i}\rangle \langle a_{i}|\rho |b_{j}\rangle .$$

The KD quasiprobability distribution satisfies several of Kolmogorov’s axioms for joint probability distributions, i.e.,

(2) $$\sum_{i,j}K_{i,j}\left(\rho \right)=1,\;\sum_{i}K_{i,j}\left(\rho \right)=\langle b_{j}|\rho |b_{j}\rangle ,\;\sum_{j}K_{i,j}\left(\rho \right)=\langle a_{i}|\rho |a_{i}\rangle .$$

It is evident that the standard KD quasiprobability distribution depends on the two bases ${\left\{|a_{i}\rangle \right\}}_{i=1}^{n}$ and ${\left\{|b_{j}\rangle \right\}}_{j=1}^{n}$ within the same Hilbert space H. However, when examining the evolution of states between two quantum systems, it raises the question of whether we can define a similar quasiprobability distribution, analogous to that in Equation (1). This consideration prompts us to introduce the concept of temporal KD quasiprobability distribution under two-time dynamics.

**Figure 1:**
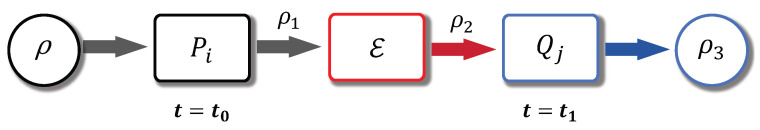
A schematic diagram of the TPSM scheme is shown. At the initial time $t=t_{0}$, the measurement $P_{i}$ is conducted on the state $\rho \in S(H_{A})$. Subsequently, the post-measurement state $\rho_{1}$ is subjected to the quantum channel E, transforming it into the state $\rho_{2}\in S\left(H_{B}\right)$. Ultimately, the measurement $Q_{j}$ is performed on $\rho_{2}$ at time $t=t_{1}$, resulting in the post-measurement state $\rho_{3}$.

Define $P={\left\{P_{i}\right\}}_{i=1}^{n}$ and $Q={\left\{Q_{j}\right\}}_{j=1}^{n}$ as two sets of projective measurements on $H_{A}$ and $H_{B}$, respectively. The operators $P_{i}$ and $Q_{j}$ represent the spectral projections associated with the observables *P* and *Q*, which can be expressed through their spectral decompositions as follows:

(3) $$P=\sum_{i=1}^{n}p_{i}P_{i},\;Q=\sum_{j=1}^{n}q_{j}Q_{j},$$

where $p_{i}$ and $q_{j}$ are eigenvalues with respect to the observables *P* and *Q*. A two-point sequential measurement (TPSM) scenario consists of the 4-tuple $\left\{\rho ,\left\{P_{i}\right\},E,\left\{Q_{j}\right\}\right\}$. First, the system *A* is prepared in the initial state $\rho \in S(H_{A})$. A projective measurement P is then performed on system *A*, after which the post-measurement state undergoes a quantum channel E∈CPTP(A,B). Finally, another projective measurement Q is performed on system *B*. The entire process of the TPSM scheme is illustrated in Figure 1.

The conditional probability of sequentially obtaining outcomes $p_{i},\;q_{j}$ associated with the measurement operators $P_{i}$ and $Q_{j}$ can be expressed as

(4) $$P\left(q_{j}|p_{i}\right)=\mathrm{Tr}\left(E\left(\rho_{1}\right)Q_{j}\right)=\frac{\mathrm{Tr}[E\left(P_{i}\rho P_{i}\right)Q_{j}]}{\mathrm{Tr}(\rho P_{i})},$$

where $\rho_{1}=P_{i}\rho P_{i}/\mathrm{Tr}\left(\rho P_{i}\right)$ denotes the state after measurement obtained via the operator $P_{i}$. The Lüders–von Neumann distribution [26], represented by the joint probability distribution,

(5) $${\left\{P_{i,j}\left(\rho \right)=\mathrm{Tr}\left[E\left(P_{i}\rho P_{i}\right)Q_{j}\right]\right\}}_{i,j},$$

encapsulates the probability of observing measurement outcomes $p_{i}$ followed by $q_{j}$, when a system initially described by the state $\rho \in S(H_{A})$ undergoes transformation through the channel E between successive measurements [22].

From a different perspective, quasiprobability provides an alternative method for describing the correlations between two incompatible observables in a quantum state. Consequently, the quasiprobability distribution of $p_{i}$ and $q_{j}$ can likewise be expressed as

(6) $${\left\{K_{i,j}\left(\rho \right)=Tr\left[E\left(\rho P_{i}\right)Q_{j}\right]\right\}}_{i,j},$$

which is referred to as the *temporal KD quasiprobability distribution* [23] under two-time dynamics. In the following, the temporal KD quasiprobability distribution we refer to is given by Equation (6) to avoid any confusion. Assume the quantum systems $H_{A}$ and $H_{B}$ are timelike-separated, where $H_{A}$ corresponds to a system at a time labeled by $t=t_{0}$, and $H_{B}$ corresponds to a system at a time labeled by $t=t_{1}$ with $t_{0}<t_{1}$. The temporal KD quasiprobability distribution relates to the distinct times $t_{0}$ and $t_{1}$, as well as the quantum systems $H_{A}$ and $H_{B}$, in which the measurements P and Q are performed.

Leveraging the property of Hilbert–Schmidt adjoint $E^{*}$, Equation (6) can be reformulated in the Heisenberg picture as

(7) $${\left\{K_{i,j}\left(\rho \right)=Tr\left[\rho P_{i}E^{*}\left(Q_{j}\right)\right]\right\}}_{i,j},$$

where $Q^{\prime }={\left\{E^{*}\left(Q_{j}\right)\right\}}_{j=1}^{n}$ denotes a POVM, since

(8) $$\sum_{j=1}^{n}E^{*}\left(Q_{j}\right)=E^{*}\left(\sum_{j=1}^{n}Q_{j}\right)=E^{*}\left(1\right)=1,\;0⩽E^{*}\left(Q_{j}\right)⩽1.$$

Consequently, the value of $K_{i,j}\left(\rho \right)$ can be derived from sequential measurements P and $Q^{\prime }$ performed on system *A*, independent of system *B*. It is important to note that while Equations (6) and (7) yield identical quasiprobability distributions, the underlying physical processes for acquiring these quasiprobabilities differ significantly. In the former case, the measurements P and Q are conducted on distinct systems *A* and *B*, whereas in the latter, the measurements P and $Q^{\prime }$ are performed on the same system *A*.

Analogously to the standard KD quasiprobability distribution, the marginals of $K_{i,j}\left(\rho \right)$ adhere to the Born rule:

(9) $$\sum_{i,j}K_{i,j}\left(\rho \right)=1,\;\sum_{i}K_{i,j}\left(\rho \right)=Tr\left[E\left(\rho \right)Q_{j}\right],\;\sum_{j}K_{i,j}\left(\rho \right)=Tr(\rho P_{i}),$$

where ${\left\{Tr[E\left(\rho \right)Q_{j}]\right\}}_{j=1}^{n}$ is the probability distribution derived from the measurement Q applied to the state E(ρ).

The mathematical equivalence of Equations (6) and (7) arises from our focus on the evolution of time from system *A* to system *B* in this work. Indeed, the temporal and spatial KD quasiprobability distributions differ significantly. For example, the two-time doubled temporal KD quasiprobability distribution is given by [23]

(10) $$K_{i,i^{\prime },j,j^{\prime }}\left(\rho \right)=Tr\{P_{i^{\prime }}\left[E\left(P_{i}\rho Q_{j}\right)\right]Q_{j^{\prime }}\},$$

which can be rewritten in the Heisenberg picture as

(11) $$K_{i,i^{\prime },j,j^{\prime }}\left(\rho \right)=Tr\left[P_{i}\rho Q_{j}E^{*}\left(Q_{j^{\prime }}P_{i^{\prime }}\right)\right],$$

where $i,i^{\prime },j,j^{\prime }\in \left\{1,2,\cdots ,n\right\}$. It is worth noting that

(12) $$\sum_{i^{\prime },j^{\prime }}E^{*}\left(Q_{j^{\prime }}P_{i^{\prime }}\right)=E^{*}\left(\sum_{i^{\prime },j^{\prime }}Q_{j^{\prime }}P_{i^{\prime }}\right)=E^{*}\left(1\right)=1.$$

However, the set ${\left\{E^{*}\left(Q_{j^{\prime }}P_{i^{\prime }}\right)\right\}}_{i^{\prime },j^{\prime }}$ does not constitute a POVM, as $E^{*}\left(Q_{j^{\prime }}P_{i^{\prime }}\right)$ is not generally positive semi-definite. Therefore, Equation (11) does not represent a spatial KD quasiprobability distribution.

The temporal KD quasiprobability distribution intrinsically captures not only information about the state but also the dynamic E, which is closely related to the correlation functions between two events [27,28]. While correlation functions between events at different times resemble joint probability distributions for the eigenvalues of the observables, they are generally neither real nor positive. The standard definition of a temporal correlation function between two events described by projectors $P_{i}^{t_{0}}$ and $Q_{j}^{t_{1}}$ is

(13) $$Q_{KD}\left(p_{i},q_{j}\right)=Tr\left(\rho P_{i}^{t_{0}}Q_{j}^{t_{1}}\right),$$

which coincides with the standard KD quasiprobability distribution. In this context, the superscripts $t_{0}$ and $t_{1}$ denote the specific times at which the measurements are taken. If a quantum channel E exists that characterizes the system’s dynamic over the interval $\left[t_{0},t_{1}\right]$, the temporal correlation function can be represented as follows:

(14) $${\tilde{Q}}_{KD}\left(p_{i},q_{j}\right)=Tr\left[\rho P_{i}^{t_{0}}E^{*}\left(Q_{j}^{t_{1}}\right)\right]=Tr\left[E\left(\rho P_{i}^{t_{0}}\right)Q_{j}^{t_{1}}\right],$$

which corresponds to the temporal KD quasiprobability distribution in this work. For brevity, we will omit the superscripts $t_{0}$ and $t_{1}$ for the projectors $P_{i}$ and $Q_{j}$ in the subsequent discussion.

## 3. Nonclassicality Measures for the Temporal KD Quasiprobability Distribution

The negative and imaginary parts of the KD quasiprobability distribution can be interpreted as nonclassical reflections, which have been shown to provide quantum advantages in a range of quantum information-processing tasks, such as quantum metrology [10,11], weak value amplification [29,30,31], proofs of contextuality [32,33,34], and quantum state tomography [35,36,37]. The characterization of nonclassicality is of paramount importance and has garnered significant attention. A widely recognized measure of KD nonclassicality is the difference between the total of the absolute values of $K_{i,j}\left(\rho \right)$ and 1, as introduced in the framework of information scrambling [38]. A sufficient condition for KD positive states has been established, along with two distinct measures to quantify the negativity and nonreality of a KD quasiprobability distribution [14]. Furthermore, it has been demonstrated that the support uncertainty can also serve as another sufficient condition for the nonclassicality of KD quasiprobability distributions [15,16]. In an analogous manner to various coherence [39,40] and entanglement measures [41,42], the diverse measures of KD nonclassicality significantly enhance our comprehension of this peculiar phenomenon in quantum mechanics.

The overall nonclassicality of a KD quasiprobability distribution is characterized by its negativity and imaginary components. By integrating Equations (5) and (6), we are led to define a measure as

(15) $$M\left(P,Q;\rho \right):=M_{Re}\left(P,Q;\rho \right)+M_{Im}\left(P,Q;\rho \right),$$

where

(16) $$M_{Re}\left(P,Q;\rho \right)=\sum_{i,j}\left|\frac{1}{2}\mathrm{Tr}\left[E\left(\left\{\rho ,P_{i}\right\}\right)Q_{j}\right]-\mathrm{Tr}\left[E\left(P_{i}\rho P_{i}\right)Q_{j}\right]\right|$$

and

(17) $$M_{Im}\left(P,Q;\rho \right)=\frac{1}{2}\sum_{i,j}|\mathrm{Tr}\left[E\left(\left[\rho ,P_{i}\right]\right)Q_{j}\right]|$$

quantify the nonclassicality of the real and imaginary parts of the temporal KD quasiprobability distribution, respectively. In this context, the symbols {·,·} and [·,·] are employed to denote the anticommutator and the commutator of two matrices, respectively. The nonclassicality measure given by Equation (15) quantify the difference between the temporal KD quasiprobability distribution and the Lüders–von Neumann distribution in the framework of the TPSM scenario. The following proposition outlines the fundamental properties of M(P,Q;ρ); see the proof in Appendix A.

**Proposition** **1.** *Let $P={\left\{P_{i}\right\}}_{i=1}^{n}$ and $Q={\left\{Q_{j}\right\}}_{j=1}^{n}$ be two sets of projective measurements. Then, we have*

*(i)*  *Faithfulness: M(P,Q;ρ)=0 if and only if the temporal KD quasiprobability distribution equals the Lüders–von Neumann distribution, i.e., $\mathrm{Tr}\left[E\left(\rho P_{i}\right)Q_{j}\right]=\mathrm{Tr}\left[E\left(P_{i}\rho P_{i}\right)Q_{j}\right]$.*
*(ii)*  *Convexity: $M\left(P,Q;\sum_{k}c_{k}\rho_{k}\right)⩽c_{k}\sum_{k}M\left(P,Q;\rho_{k}\right)$, where $\left\{c_{k}\right\}$ is a probability distribution satisfying $c_{k}⩾0$ and $\sum_{k}c_{k}=1$.*
*(iii)*  *Noncommutativity witness: If M(P,Q;ρ)>0, then there is a choice of the indices i and j, for which $P_{i}$ does not commute with ρ and $E^{*}\left(Q_{j}\right)$, where $E^{*}$ denotes the Hilbert–Schmidt adjoint of E.*
*(iv)*  *Monotonic decrease under decoherence: The decoherence channel is represented as $D_{s}=\left(1-s\right)1+sD$, where s∈[0,1], with 1 denoting the identity channel and D signifying the completely dephasing channel defined by $D\left(\rho \right)=\sum_{i}P_{i}\rho P_{i}$. Consequently, it follows that $M(P,Q;D_{s}\left(\rho \right))⩽M\left(P,Q;\rho \right)$.*

Building on the findings from Proposition 1, a pertinent question arises: If M(P,Q;ρ)=0 holds for any $\rho \in S(H_{A})$, does it imply that $P_{i}$ commutes with $E^{*}\left(Q_{j}\right)$ for all pairs (i,j)? We establish an affirmative answer; see the proof of Theorem 1 in Appendix B.

**Theorem** **1.** *Let $P={\left\{P_{i}\right\}}_{i=1}^{n}$ and $Q={\left\{Q_{j}\right\}}_{j=1}^{n}$ denote two projective measurements on the Hilbert spaces $H_{A}$ and $H_{B}$, respectively. Then, the condition M(P,Q;ρ)=0 for any $\rho \in S(H_{A})$ is equivalent to $[P_{i},E^{*}\left(Q_{j}\right)]=0$ for all indices i and j.*

Indeed, the validity of Theorem 1 can be extended to the case where P and Q are POVMs, provided that each $E^{*}\left(Q_{j}\right)$ possesses distinct eigenvalues. The physical meaning of this condition is that the observable $E^{*}\left(Q_{j}\right)$ is non-degenerate for each *j*. This generalization to POVMs is closely related to the Lüders theorem for unsharp quantum measurements, which relies on the condition that the eigenvalues of one observable are distinct [43]. Moreover, if the POVM $\tilde{P}$ consists of only two elements, i.e., $\tilde{P}=\left\{{\tilde{P}}_{1},{\tilde{P}}_{2}\right\}$, then the generalized Lüders theorem remains valid even in the case where the observable has degenerate eigenvalues [43]. However, the issue of degenerate eigenvalues in other cases remains unresolved. The temporal KD quasiprobability distribution and the associated nonclassicality measures, as described in Equations (6) and (15), depend on the choice of various channels, as demonstrated by the following two illustrative examples.

**Example** **1.** *Provided that E is the depolarizing channel given by*

(18) $$E\left(X\right)=\left(1-\eta \right)X+\eta \mathrm{Tr}(X)\cdot \frac{1}{n},$$

*where η∈[0,1] is the depolarization parameter and X∈A. Consequently, the temporal KD quasiprobability distribution is given by*

(19) $$K_{i,j}\left(\rho \right)=\left(1-\eta \right)\mathrm{Tr}\left(\rho P_{i}Q_{j}\right)+\frac{\eta }{n}\mathrm{Tr}(\rho P_{i}),$$

*which can be interpreted as a weighted average of the standard KD quasiprobability distribution and the probability distribution ${\left\{\mathrm{Tr}(\rho P_{i})\right\}}_{i=1}^{n}$ derived from the Born rule. Meanwhile, the nonclassicality measure defined by Equation (15) is found to be*

(20) M(P,Q;ρ)=(1−η)∑i,j(|ReTr(PiQjρ)−Tr(PiρPiQj)|+|ImTr(PiQjρ)|),

*where the symbols Re and Im denote the real and imaginary parts, respectively. When η=0, the temporal KD quasiprobability distribution simplifies to the standard KD quasiprobability distribution. Furthermore, the nonclassicality measure defined in Equation (20) corresponds to the KD nonclassicality measure discussed in ref. [44] when η=0.*

In fact, the temporal KD quasiprobability distribution can also be viewed as a generalized phase-space representation of a quantum state [27,28]. In this context, the standard KD quasiprobability distribution may be interpreted in two distinct ways [23]: (i) as an equal-time phase-space representation of a given state, or (ii) as a two-time temporal KD quasiprobability distribution with the evolution given by the identity channel (E=1). Consequently, the KD nonclassicality measure discussed in ref. [44] is a special case of the temporal KD nonclassicality measure presented in this work. Our temporal KD nonclassicality measure may prove useful in various quantum information-processing tasks involving quantum processes. For example, the temporal correlation function [27,28] between two events can be expressed through the temporal KD quasiprobability distribution, whose nonclassicality can be characterized using the temporal KD nonclassicality measure developed here. However, the KD nonclassicality measure defined in ref. [44] is not suitable for this scenario. In other words, the applicability of our temporal KD nonclassicality is much broader than that of the measure in ref. [44].

**Example** **2.** *Let E denote the discard-and-prepare channel given by E(X)=Tr(X)σ for any X∈A, where $\sigma \in S(H_{B})$. Then, we have*

(21) $$K_{i,j}\left(\rho \right)=\mathrm{Tr}\left(\rho P_{i}\right)\cdot \mathrm{Tr}\left(\sigma Q_{j}\right).$$

*While the Lüders–von Neumann distribution defined by Equation (5) can be represented as*

(22) $$P_{i,j}\left(\rho \right)=\mathrm{Tr}\left[E\left(P_{i}\rho P_{i}\right)Q_{j}\right]=\mathrm{Tr}\left(\rho P_{i}\right)\cdot \mathrm{Tr}\left(\sigma Q_{j}\right),$$

*utilizing the property that $P_{i}$ and $Q_{j}$ are rank-one projections. Thus, we conclude that M(P,Q;ρ)=0 in this specific scenario.*

For a specific TPSM scheme $\left\{\rho ,\left\{P_{i}\right\},E,\left\{Q_{j}\right\}\right\}$, it has been shown that the temporal KD nonclassicality M(P,Q;ρ) is bounded. See the proof of Proposition 2 in Appendix C.

**Proposition** **2.** *The upper bound of the temporal KD nonclassicality satisfies*

(23) $$M\left(P,Q;\rho \right)⩽\sqrt{2n\left(n-1\right)C_{l_{2}}\left(\rho \right)}\;,$$

*where $C_{l_{2}}\left(\rho \right)=\sum_{k\neq i}{\left|\rho_{ki}\right|}^{2}$ is the $l_{2}$-norm coherence of the state ρ with respect to the reference basis ${\left\{|p_{i}\rangle \right\}}_{i=1}^{n}$ [39], and $\rho_{ki}:=\langle p_{k}|\rho |p_{i}\rangle$ denotes the matrix element of ρ. Moreover, the upper bound is achieved if and only if both $E^{*}\left(Q_{j}\right)$ and ρ are maximally coherent states, i.e., $|\langle p_{i}E^{*}\left(Q_{j}\right)p_{k}\rangle \left|=\right|\rho_{ki}|=1/n$ for all i,j,k∈{1,2,⋯,n}.*

The maximum value of upper bound in Proposition 2 is $\left(n-1\right)\sqrt{2}$, given that $C_{l_{2}}\left(\rho_{max}\right)=\left(n-1\right)/n$, where $\rho_{max}$ denotes the maximally coherent state. Consequently, as the dimension of the system *n* increases, the upper limit of temporal KD nonclassicality rises. This upper bound also implies that lower coherence of ρ will result in reduced temporal KD nonclassicality. Nonetheless, there may not be a direct quantitative relationship between the temporal KD nonclassicality and the coherence of the initial state. This phenomenon can be elucidated within the resource theory of imaginarity [45]. In this framework, the free states are identified as real states, while real operations correspond to free operations. We initially treat $\rho^{\left(1\right)}=\left(\rho_{ij}^{\left(1\right)}\right)$ as a real state, where $\rho_{ij}^{\left(1\right)}\in R$ denotes the matrix element. For the state $\rho^{\left(2\right)}=\left(\rho_{ij}^{\left(1\right)}e^{i\theta_{ij}}\right)$, with $\theta_{ij}\in R$, it follows that $C_{l_{2}}\left(\rho^{\left(1\right)}\right)=C_{l_{2}}\left(\rho^{\left(2\right)}\right)$. However, there are cases where $M(P,Q;\rho^{\left(1\right)})$ does not equal $M(P,Q;\rho^{\left(2\right)})$ for real operation E and real measurements P, Q. Specifically, we find that $M_{Im}\left(P,Q;\rho^{\left(1\right)}\right)=0$. We then provide a specific example that achieves the upper bound in Proposition 2.

**Example** **3.** *Let $\mathrm{Let}\;\{|p_{1}\rangle =|0\rangle ,|p_{2}\rangle =|1\rangle \}$ and $\left\{|q_{1}\rangle =\left(|0\rangle +i|1\rangle \right)/\sqrt{2},\;|q_{2}\rangle =\left(|0\rangle -i|1\rangle \right)/\sqrt{2}\right\}$ denote two orthogonal bases in a two-dimensional Hilbert space, where the notation i denotes the imaginary unit. For the maximally coherent state ρ=|ψ⟩⟨ψ| with $|\psi \rangle =\left(|0\rangle +e^{i\varphi }|1\rangle \right)/\sqrt{2}$, it is straightforward to compute the values of Equations (16) and (17) when E is the identity channel, i.e.,*

(24) $$M_{Re}\left(P,Q;\rho \right)=|\sin\varphi |,\;M_{Im}\left(P,Q;\rho \right)=\left|\cos\varphi \right|,\;\varphi \in \left[0,\pi \right].$$

*In this example, the maximum value presented in Proposition 2 is $\sqrt{2}$, which is achieved at φ=π/4 or 3π/4. The corresponding Lüders–von Neumann distribution $P_{i,j}\left(\rho \right)$ and temporal KD quasiprobability distribution $K_{i,j}\left(\rho \right)$ when φ=π/4 are shown in Table 1.*

## 4. Temporal KD Nonclassicality, Uncertainty, and Causal Correlations

### 4.1. Temporal KD Nonclassicality and Uncertainty

In this subsection, we derive two uncertainty relations related to the temporal KD nonclassicality M(P,Q;ρ). The first relation serves as a direct generalization of the corresponding result presented in ref. [44], whereas the second relation provides a new feature, demonstrating that M(P,Q;ρ) is constrained from below by the measurement disturbance caused by the measurement P.

#### 4.1.1. Connection with the Schrödinger Uncertainty Relation

The temporal KD nonclassicality M(P,Q;ρ), as defined in Equation (15), can be interpreted as a measure based on the $l_{1}$-norm. Alternatively, we can describe the nonclassicality of the temporal KD quasiprobability distribution using a different approach. For example, when employing the $l_{2}$-norm, the nonclassicality measure associated with the temporal KD quasiprobability distribution is expressed as

(25) $$\tilde{M}\left(P,Q;\rho \right)=\sum_{i,j}{\left({\left|\frac{1}{2}\mathrm{Tr}\left[E\left(\left\{\rho ,P_{i}\right\}\right)Q_{j}\right]-\mathrm{Tr}\left[E\left(P_{i}\rho P_{i}\right)Q_{j}\right]\right|}^{2}+{\left|\frac{1}{2}\mathrm{Tr}\left[E\left(\left[\rho ,P_{i}\right]\right)Q_{j}\right]\right|}^{2}\right)}^{1/2}.$$

The two temporal KD nonclassicality measures, defined by Equations (15) and (25), are mathematically equivalent, as demonstrated by the inequalities $\tilde{M}\left(P,Q;\rho \right)⩽M\left(P,Q;\rho \right)⩽\sqrt{2}\tilde{M}\left(P,Q;\rho \right)$. In the following, we will establish that the quantity $\tilde{M}\left(P,Q;\rho \right)$ acts as the lower bound for the Schrödinger uncertainty relation when appropriate states and observables are chosen.

The well-known Schrödinger uncertainty relation [46] states that for two noncommuting observables X,Y and a state σ=|ϕ⟩⟨ϕ|, the following inequality holds:

(26) $$\Delta {\left(X\right)}^{2}\cdot \Delta {\left(Y\right)}^{2}⩾{\left|\frac{1}{2}\langle \phi |\left\{X,Y\right\}|\phi \rangle -\langle \phi |X|\phi \rangle \langle \phi |Y|\phi \rangle \right|}^{2}+{\left|\frac{1}{2}\langle \phi |\left[X,Y\right]|\phi \rangle \right|}^{2},$$

where $\Delta {\left(X\right)}^{2}=\mathrm{Tr}\left(\rho X^{2}\right)-\mathrm{Tr}{\left[\left(\rho X\right)\right]}^{2}$ is the variance, similar to $\Delta {\left(Y\right)}^{2}$. We also observe that

(27) $$\mathrm{Tr}[E\left(P_{i}\rho P_{i}\right)Q_{j}]=\mathrm{Tr}\left(\rho P_{i}\right)\cdot \mathrm{Tr}\left[E^{*}\left(Q_{j}\right)P_{i}\right],$$

where we utilize the property that $P_{i}$ and $Q_{j}$ are projections of rank one.

By examining the right-hand sides of Equations (25)–(27), we can derive the following uncertainty relation by setting X=ρ, $Y=E^{*}\left(Q_{j}\right)$, and $\sigma =|p_{i}\rangle \langle p_{i}|$, i.e.,

(28) $$\Delta \left(\rho \right)\cdot \Delta \left(E^{*}\left(Q_{j}\right)\right)⩾\tilde{M}\left(P_{i},Q_{j};\rho \right),$$

where

(29) $$\tilde{M}\left(P_{i},Q_{j};\rho \right)={\left({\left|\frac{1}{2}\mathrm{Tr}\left[E\left(\left\{\rho ,P_{i}\right\}\right)Q_{j}\right]-\mathrm{Tr}\left[E\left(P_{i}\rho P_{i}\right)Q_{j}\right]\right|}^{2}+{\left|\frac{1}{2}\mathrm{Tr}\left[E\left(\left[\rho ,P_{i}\right]\right)Q_{j}\right]\right|}^{2}\right)}^{1/2}.$$

In this context, both ρ and $E^{*}\left(Q_{j}\right)$ are treated as observables that are constrained by the identity operator 1, which are identified as quantum effects [47]. Equation (28) establishes that the temporal KD nonclassicality $\tilde{M}\left(P_{i},Q_{j};\rho \right)$, associated with each pair of $P_{i}$ and $Q_{j}$, is equivalent to the lower bound of the product of the standard deviations of the measurements of ρ and $E^{*}\left(Q_{j}\right)$ for a specific quantum state $\left|p_{i}\rangle \langle p_{i}\right|$. By summing over all indices *i* and *j* in the aforementioned tradeoff relation expressed in Equation (28), we derive

(30) $$\sum_{i,j}\Delta \left(\rho \right)\cdot \Delta \left(E^{*}\left(Q_{j}\right)\right)⩾\tilde{M}\left(P,Q;\rho \right)⩾\frac{M(P,Q;\rho )}{\sqrt{2}},$$

which indicates that the temporal KD nonclassicality can be regarded as the lower bound of the uncertainty relation.

#### 4.1.2. A State-Dependent Uncertainty Relation for the Temporal KD Nonclassicality

As demonstrated in Proposition 1, the quantity M(P,Q;ρ) measures the difference between the temporal KD quasiprobability distribution and the Lüders–von Neumann distribution. This difference can be interpreted as the cumulative effect of these discrepancies across all indices i,j. Therefore, we will concentrate on each term in Equations (16) and (17) with the aim of establishing a new uncertainty relation.

Let us define the following two quantities:

(31) MRe(Pi,Qj;ρ):=ReTr[E(ρPi)Qj]−Tr[E(PiρPi)Qj],

(32) MIm(Pi,Qj;ρ):=ImTr[E(ρPi)Qj],

where the symbols Re and Im denote the real and imaginary parts, respectively. By combining Equations (31) and (32), we can obtain

(33) $$\sum_{i}\left[M_{Re}\left(P_{i},Q_{j};\rho \right)+iM_{Im}\left(P_{i},Q_{j};\rho \right)\right]=\mathrm{Tr}\left[E\left(\rho -\sum_{i}P_{i}\rho P_{i}\right)Q_{j}\right],$$

where the notation i denotes the imaginary unit, and the linearity of E is utilized.

By triangle inequality, we proceed to have

(34) $$\begin{array}{cc} \left|\mathrm{Tr}\left[E\left(\rho -\sum_{i}P_{i}\rho P_{i}\right)Q_{j}\right]\right| & ⩽\sum_{i}|\left[M_{Re}\left(P_{i},Q_{j};\rho \right)+iM_{Im}\left(P_{i},Q_{j};\rho \right)\right]| \end{array}$$

(35) $$\begin{array}{cc} \; & ⩽\sum_{i}\left\{\right|M_{Re}\left(P_{i},Q_{j};\rho \right)\left|+\right|M_{Im}\left(P_{i},Q_{j};\rho \right)\left|\right\}. \end{array}$$

It is straightforward to observe that

(36) $$\begin{array}{cc} M_{Re}\left(P,Q;\rho \right) & =\sum_{i,j}\left|M_{Re}\left(P_{i},Q_{j};\rho \right)\right|, \end{array}$$

(37) $$\begin{array}{cc} M_{Im}\left(P,Q;\rho \right) & =\sum_{i,j}\left|M_{Im}\left(P_{i},Q_{j};\rho \right)\right|, \end{array}$$

where $M_{Re}\left(P,Q;\rho \right)$ and $M_{Im}\left(P,Q;\rho \right)$ are defined in Equations (16) and (17), respectively.

By performing a summation over the index *j* on both sides of Equation (35) and applying the properties of the Hilbert-Schmidt adjoint $E^{*}$, we finally arrive at

(38) $$\begin{array}{c} M\left(P,Q;\rho \right)=M_{Re}\left(P,Q;\rho \right)+M_{Im}\left(P,Q;\rho \right)⩾\sum_{j}|\mathrm{Tr}\left[\left(\rho -I_{L}\left(\rho \right)\right)E^{*}\left(Q_{j}\right)\right]|, \end{array}$$

where $I_{L}\left(\rho \right)=\sum_{i}P_{i}\rho P_{i}$ is the Lüders transformation of the state ρ [43]. Each term on the right-hand side of Equation (38) can be interpreted as the disturbance introduced by the measurement $P={\left\{P_{i}\right\}}_{i=1}^{n}$ within a sequential measurement framework. Specifically, we first conduct the measurement $P={\left\{P_{i}\right\}}_{i=1}^{n}$ and subsequently apply the POVM $Q^{\prime }={\left\{E^{*}\left(Q_{j}\right)\right\}}_{j=1}^{n}$ on the post-measurement state $I_{L}\left(\rho \right)$. The right-hand side of Equation (38) effectively captures the $l_{1}$-norm distance between the two probability distributions $q_{1}={\left\{\mathrm{Tr}\left[\rho E^{*}\left(Q_{j}\right)\right]\right\}}_{j=1}^{n}$ and $q_{2}={\left\{\mathrm{Tr}\left[I_{L}\left(\rho \right)E^{*}\left(Q_{j}\right)\right]\right\}}_{j=1}^{n}$. The condition $q_{1}=q_{2}$ indicates that the measurement P does not affect $Q^{\prime }$, which also implies the probability derived from the measurement $Q^{\prime }$ remains unchanged regardless of whether the measurement P is conducted prior to $Q^{\prime }$. Furthermore, if the condition $q_{1}=q_{2}$ is satisfied for any ρ, it follows from the Lüders theorem [43] that $[P_{i},E^{*}\left(Q_{j}\right)]=0$. Utilizing Theorem 1, we can conclude that M(P,Q;ρ)=0 in this scenario, which further indicates that the temporal KD quasiprobability distribution simplifies to the Lüders–von Neumann distribution.

### 4.2. Temporal KD Nonclassicality and Causal Correlations

In this subsection, we aim to delineate the relationships between the temporal KD nonclassicality and causal correlations. We begin by presenting the three causal structures for two observables. Following this, we provide a brief overview of recent advancements concerning canonical states over time and PDMs to ensure the consistency of our work. Finally, we establish the links among the temporal KD nonclassicality, spatiotemporal Born rule, and spatiotemporal compatibility.

#### 4.2.1. Three Causal Structures for Two Observables

In general, there are three causal structures for two observables M∈A,N∈B, classified based on their expectation value 〈M,N〉. The expectation value 〈M,N〉 is derived from the statistics of the measurement outcomes corresponding to the measurements performed on systems *A* and *B*. We will proceed to examine these three causal structures for the two observers in order to establish their relationship with the temporal KD nonclassicality. We thus begin by revisiting the definition of the two-time expectation value, which serves to define the temporal compatibility.

**Definition** **1** ([22,48])**.** *The two-time expectation value ${\langle M,N\rangle }_{\left(E,\rho_{A}\right)}$ for the observables M∈A and N∈B, with respect to the process $\left(E,\rho_{A}\right)\in P\left(A,B\right)$, is expressed as the real number given by*

(39) $${\langle M,N\rangle }_{\left(E,\rho_{A}\right)}=\sum_{i}\lambda_{i}\mathrm{Tr}\left[E\left(M_{i}\rho_{A}M_{i}\right)N\right],\;\rho_{A}\in S\left(H_{A}\right),$$

*where the observable M can be represented through its canonical spectral decomposition as $M=\sum_{i}\lambda_{i}M_{i}$, with $M_{i}$ denoting the projector onto the eigenspace corresponding to the eigenvalue $\lambda_{i}$.*

It has been demonstrated that there is no operator $\varrho_{AB}$ such that ${\langle M,N\rangle }_{\left(E,\rho_{A}\right)}=\mathrm{Tr}\left[\varrho_{AB}\left(M⊗N\right)\right]$ for any observables M∈A and N∈B [22]. In other words, the process $\left(E,\rho_{A}\right)\in P\left(A,B\right)$ cannot be represented for arbitrary observables *M* and *N*. Nevertheless, the process $\left(E,\rho_{A}\right)$ is representable when *M* is a light-touch observable [22]. Light-touch observables are defined as those whose spectra are either {λ} or {±λ} for some λ>0, thus encompassing the Pauli observables within a wider range. Let $\left\{M_{a}\right\}$ and $\left\{N_{b}\right\}$ denote two collections of light-touch observables; consequently, the three causal structures for two observables can be categorized in the following manner.

**Definition** **2** ([21,48,49])**.**

*(i)*  ***Spatially compatible.*** *There exists a density matrix $\rho_{AB}$ such that $\langle M_{a},N_{b}\rangle =\mathrm{Tr}[\rho_{AB}\left(M_{a}⊗N_{b}\right)]$ for all a,b. Then, the expectation values are said to be spatially compatible on the joint system A⊗B. If no such $\rho_{AB}$ exists, then expectation values are said to be spatially incompatible.*
*(ii)*  ***Temporally compatible with temporal order* A→B.*** There exists a density matrix $\rho_{A}\in A$ and a quantum channel E:A→B such that $\langle M_{a},N_{b}\rangle$ equals the two-time expectation value associated with the process $\left(E,\rho_{A}\right)$, i.e.,*
  (40) $$\langle M_{a},N_{b}\rangle =\sum_{x}\lambda_{x}\mathrm{Tr}[E\left(M_{x|a}\rho_{A}M_{x|a}\right)N_{b}],$$
  *for all a,b. Here, $M_{a}=\sum_{x}\lambda_{x}M_{x|a}$ is the canonical spectral decomposition of the observable $M_{a}$, and $M_{x|a}$ corresponds to the spectral projection associated with eigenvalue $\lambda_{x}$.*
*(iii)*  ***Temporally compatible with temporal order* B→A.*** There exists a density matrix $\rho_{B}\in B$ and a quantum channel F:B→A such that $\langle M_{a},N_{b}\rangle$ equals the two-time expectation value associated with the process $\left(F,\rho_{B}\right)$, i.e.,*
  (41) $$\langle M_{a},N_{b}\rangle =\sum_{y}\mu_{y}\mathrm{Tr}[F\left(N_{y|b}\rho_{B}N_{y|b}\right)M_{a}],$$
  *for all a,b. Here, $N_{b}=\sum_{y}\mu_{y}N_{y|b}$ is the canonical spectral decomposition of the observable $N_{b}$, and $N_{y|b}$ corresponds to the spectral projection associated with eigenvalue $\mu_{y}$.*

#### 4.2.2. Spatiotemporal Born Rule

The joint probabilities arising from measurements on spacelike separated quantum systems are articulated through the Born rule. In contrast, the joint probabilities for the outcomes of sequential measurements on a quantum system generally cannot be consistently derived from the trace of a fixed operator multiplied by a tensor product of projectors. However, it has been demonstrated that by introducing a correction term to the probabilities of the TPSM scheme, one can derive a quasiprobability distribution that is uniquely represented by a bipartite operator. This operator can be interpreted as a spatiotemporal quantum state, thereby providing a spatiotemporal extension of the Born rule [26]. It is specifically established that for every TPSM scenario $\left\{\rho_{A},\left\{P_{i}\right\},E,\left\{Q_{j}\right\}\right\}$, there exists a unique operator $\varrho_{AB}\in H_{A}⊗H_{B}$ such that for any $\rho_{A}\in S\left(H_{A}\right)$, we have [26]

(42) $$Q_{i,j}\left(\rho_{A}\right)=\mathrm{Tr}\left[\varrho_{AB}\left(P_{i}⊗Q_{j}\right)\right],$$

where Qi,j(ρA)=ReKi,j(ρA) is the temporal Margenau–Hill distribution. Moreover, the operator $\varrho_{AB}$ is given by [22,26]

(43) $$\varrho_{AB}=E★\rho_{A}:=\frac{1}{2}\left\{\rho_{A}⊗1_{B},J\left[E\right]\right\},$$

where $J\left[E\right]=\sum_{i,j}|i\rangle \langle j|⊗E\left(|j\rangle \langle i|\right)$ is the Jamiołkowski matrix associated with the channel E [50] and {·,·} denotes the anticommutator. The operator $\varrho_{AB}$ is referred to as the canonical state over time that corresponds to the process $\left(E,\rho_{A}\right)\in P\left(A,B\right)$ [22]. Next, we will briefly revisit the concept of the pseudo-density matrix (PDM) to help readers understand the relationships between PDMs and canonical states over time.

We examine the following particular case. Let systems A and B each consist of *m* qubits, meaning that $\dim H_{A}=\dim H_{B}=2^{m}$. The Pauli matrices are denoted by $\sigma_{1},\sigma_{2},\sigma_{3}$, respectively, and $\sigma_{0}$ represents the identity matrix. A Pauli observable $\sigma_{\alpha }$ can be formulated as $\sigma_{\alpha }=\sigma_{\alpha_{1}}⊗\sigma_{\alpha_{2}}\cdots \sigma_{\alpha_{m}}$, where $\alpha \in {\left\{0,1,2,3\right\}}^{m}$ and each $\alpha_{k}\in \left\{0,1,2,3\right\}$ for k∈{1,2,⋯,m}. For any process $\left(E,\rho_{A}\right)\in P\left(A,B\right)$, there exists a unique Hermitian operator $\varrho_{AB}\in A⊗B$, known as the PDM corresponding to $\left(E,\rho_{A}\right)$, such that ${\langle \sigma_{\alpha },\sigma_{\beta }\rangle }_{\left(E,\rho_{A}\right)}=\mathrm{Tr}[\varrho_{AB}\left(\sigma_{\alpha }⊗\sigma_{\beta }\right)]$, where $\sigma_{\alpha }$ and $\sigma_{\beta }$ are the Pauli observables. Moreover, the operator $\varrho_{AB}$ is given by [19]

(44) $$\varrho_{AB}=\frac{1}{4^{m}}\sum_{\alpha ,\beta \in {\left\{0,1,2,3\right\}}^{m}}{\langle \sigma_{\alpha },\sigma_{\beta }\rangle }_{\left(E,\rho_{A}\right)}\sigma_{\alpha }⊗\sigma_{\beta }.$$

The PDM indeed represents a special case of the canonical state over time, as it can be expressed through a channel E such that $\varrho_{AB}$ takes the form given in Equation (43), where $\rho_{A}={\mathrm{Tr}}_{B}\left[\varrho_{AB}\right]$ denotes the reduced state of system *A* [20,21]. Additionally, it has been shown that the canonical states over time serve as the unique operator representation for two-time expectation values constrained to light-touch observables, thereby offering a suitable extension of Pauli observables across all dimensions [22].

The core idea of the spatiotemporal Born rule is to establish a unique operator representation for the two-time expectation values of any observables, rather than being limited to light-touch observables, thereby yielding a spatiotemporal extension of the Born rule. A spatiotemporal Born rule is then said to exist for the Lüders–von Neumann distribution with respect to the process $\left(E,\rho_{A}\right)\in P\left(A,B\right)$ if and only if there exists an operator $\varrho_{AB}$ given by Equation (43) such that for all TPSM scenarios [26],

(45) $$\mathrm{Tr}[\varrho_{AB}\left(P_{i}⊗Q_{j}\right)]=\mathrm{Tr}\left[E\left(P_{i}\rho P_{i}\right)Q_{j}\right].$$

Operationally, Equation (45) indicates the expectation value 〈P,Q〉 is both spatially and temporally compatible when $\varrho_{AB}$ is a density matrix. Thus, it is sufficient for $\varrho_{AB}$ to be positive semi-definite, as we have ${\mathrm{Tr}}_{\varrho AB}=1$. This observation can be summarized as follows.

**Proposition** **3.** *If $\varrho_{AB}=E★\rho_{A}$ is positive semi-definite, then the existence of a spatiotemporal Born rule for the Lüders–von Neumann distribution concerning the process $\left(E,\rho_{A}\right)\in P\left(A,B\right)$ implies that the expectation value 〈P,Q〉 is both spatially and temporally compatible with the temporal order E:A→B.*

#### 4.2.3. Connection with the Temporal KD Nonclassicality

From Proposition 3, it can be deduced that the existence of the spatiotemporal Born rule for Lüders–von Neumann distribution is closely connected to the negativity of the temporal KD quasiprobability distribution. We thus have the following result; see the proof in Appendix D.

**Theorem** **2.** *A spatiotemporal Born rule exists for all Lüders–von Neumann distributions with respect to the process $\left(E,\rho_{A}\right)\in P\left(A,B\right)$ if and only if $M_{Re}\left(P,Q;\rho_{A}\right)=0$. Furthermore, if E is injective, we must have $\rho_{A}=1/n$.*

It is worth noting that the scenario in which the channel E is not injective remains unresolved. We propose the conjecture that E should be classified as a discard-and-prepare channel, as illustrated in Example 2. By integrating Proposition 3 and Theorem 2, we can deduce that if 〈P,Q〉 is either spatially or temporally incompatible, then $M_{Re}\left(P,Q;\rho_{A}\right)>0$. This indicates that the negativity of the temporal KD quasiprobability distribution serves as a necessary condition for spatiotemporal incompatibility. However, whether we can impose additional conditions alongside the negativity of the temporal KD quasiprobability distribution to precisely determine the causal structures remains a topic for further investigation.

When considering the light-touch observables, it has been demonstrated that the bipartite state τ∈A⊗B is separable, then there exists a channel E such that $\tau =E★\rho_{A}$ [48], where $\rho_{A}={\mathrm{Tr}}_{B}\left[\tau \right]$ is the reduced state of system *A*. Building on this observation, we explore the relationship between bipartite separability and the temporal KD quasiprobability distribution; see the proof of Theorem 3 in Appendix D.

**Theorem** **3.** *If the bipartite state τ∈A⊗B is separable, and the light-touch observable P∈A along with the arbitrary observable Q∈B can be expressed through their spectral decompositions in Equation (3), then there exists a channel E such that ReTr[E(ρAPi)Qj]⩾0, where $\rho_{A}={\mathrm{Tr}}_{B}\left[\tau \right]$ denotes the reduced state of system A.*

Indeed, the temporal compatibility implies that 〈P,Q〉=ReTr[E(ρAP)Q] for the light-touch observable P∈A. See the proof of Corollary 1 in Appendix D.

**Corollary** **1.** *Let P∈A be a light-touch observable, and let Q∈B denote an arbitrary observable. Moreover, if the expectation value 〈P,Q〉 is temporally compatible with temporal order E:A→B, then it follows that ReTr[E(ρAP)Q]=∑ipiTr[E(PiρAPi)Q]=〈P,Q〉, where P has spectral decomposition as $P=\sum_{i=1}^{n}p_{i}P_{i}$, and $\rho_{A}\in S\left(H_{A}\right)$.*

**Figure 2:**
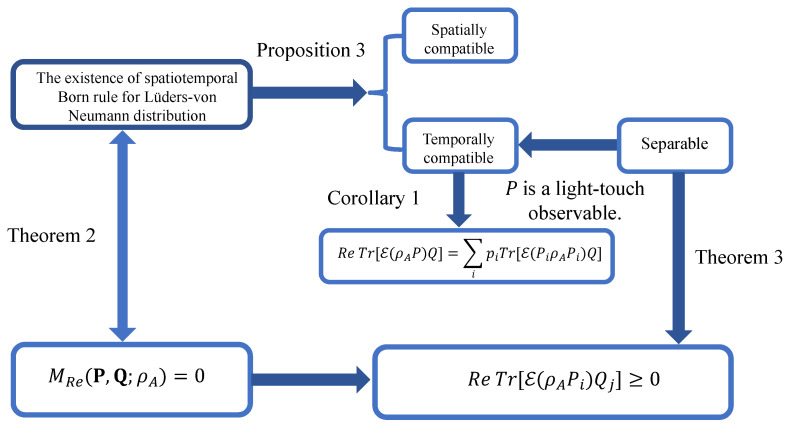
The interplay among the temporal KD nonclassicality, spatiotemporal Born rule, and spatiotemporal compatibility is shown.

We note that ref. [48] has demonstrated that separability indicates temporal compatibility. By integrating Proposition 3 and Corollary 1 with Theorems 2 and 3, we can illustrate the interplay among temporal KD nonclassicality, the spatiotemporal Born rule, and spatiotemporal compatibility in Figure 2. However, the reverse is not generally true in Figure 2. For example, temporal compatibility does not necessarily lead to separability [48]. Moreover, Theorem 3 and Corollary 1 require that *P* is a light-touch observable, while Theorem 2 and Proposition 3 do not impose this condition. Additionally, if we consider *Q* as a light-touch observable, we can construct a similar diagram by examining the temporal order represented by F:B→A.

## 5. Conclusions

In this work, we deeply investigate the evolution of the KD quasiprobability distribution under two-time dynamics. Specifically, we present a nonclassicality measure based on $l_{1}$-norm for the temporal KD quasiprobability distribution and illustrate that this measure exhibits several desirable characteristics typical of a nonclassicality measure. The upper bound of this nonclassicality is closely associated with both the dimensionality and $l_{2}$-norm coherence of the initial state, regardless of the choice of channel. Subsequently, we put forward two uncertainty relations concerning the temporal KD nonclassicality M(P,Q;ρ). The second of these relations implies that M(P,Q;ρ) is lower-bounded by the measurement disturbance caused by measurement P. We finally establish the relationship among the temporal KD nonclassicality, spatiotemporal Born rule, and spatiotemporal compatibility.

Our findings indicate that the negativity of the temporal KD quasiprobability distribution is intrinsically linked to spatiotemporal correlations, thereby providing a new unified framework for understanding quantum correlations. However, our study represents only an initial step, and several aspects require further investigation. One of the most critical areas is precisely identifying causal structures using the properties of the temporal KD quasiprobability distribution. Additionally, our work focuses solely on the two-time dynamics of the KD quasiprobability distribution. The multi-time dynamics of the KD quasiprobability distribution are likely connected to multipartite quantum states over time [51] and more general causal structures [52,53].

The temporal KD nonclassicality may have potential applications in quantum information tasks involving temporal quantum processes. For example, in postselected metrology, the encoded pure state may pass through a noisy channel due to decoherence effects. Consequently, it becomes essential to estimate the quantum Fisher information (QFI) of a mixed state, which can be addressed using Krylov shadow tomography [54]. The QFI of the postselected state is likely expressible in terms of the temporal KD quasiprobability distribution. Additionally, the negativity of the temporal KD quasiprobability distribution might enhance the postselected QFI, similar to the findings in ref. [10]. Furthermore, the out-of-time-ordered correlator has been shown to correspond to a moment of a summed quasiprobability [18], which can also be regarded as a special form of a multi-time temporal KD quasiprobability distribution. In summary, the temporal KD quasiprobability distribution is rich in context, and all the intriguing directions mentioned above warrant further exploration.

## Figures and Tables

**Table 1:** The $P_{i,j}\left(\rho \right)$ and $K_{i,j}\left(\rho \right)$ in Example 3 are tabulated, where φ=π/4.

	$P_{i,j}\left(\rho \right)$		$K_{i,j}\left(\rho \right)$	
Basis	$\left|p_{1}\right\rangle$	$\left|p_{2}\right\rangle$	$\left|p_{1}\right\rangle$	$\left|p_{2}\right\rangle$
$\left|q_{1}\right\rangle$	$\frac{1}{4}$	$\frac{1}{4}$	$\frac{2+\sqrt{2}\left(1-i\right)}{8}$	$\frac{2+\sqrt{2}\left(1+i\right)}{8}$
$\left|q_{2}\right\rangle$	$\frac{1}{4}$	$\frac{1}{4}$	$\frac{2-\sqrt{2}\left(1-i\right)}{8}$	$\frac{2-\sqrt{2}\left(1+i\right)}{8}$

## Data Availability

No new data were created or analyzed in this study.

## Appendix A

**Proof of Proposition** **1.** (i) The expression in Equation (15) indicates that M(P,Q;ρ)=0 if and only if both $M_{Re}\left(P,Q;\rho \right)=0$ and $M_{Im}\left(P,Q;\rho \right)=0$. This condition is also equivalent to the statements ReTr[E(ρPi)Qj]=Tr[E(PiρPi)Qj] and ImTr[E(ρPi)Qj]=0. In other words, the temporal KD quasiprobability distribution equals the Lüders–von Neumann distribution. (ii) From Equation (15), it can be inferred that

(A1) $$\begin{array}{cc} M(P,Q;\rho ) & =\sum_{i,j}\left|\frac{1}{2}\mathrm{Tr}\left[E\left(\left\{\rho ,P_{i}\right\}\right)Q_{j}\right]-\mathrm{Tr}\left[E\left(P_{i}\rho P_{i}\right)Q_{j}\right]\right|+\frac{1}{2}\sum_{i,j}|\mathrm{Tr}\left[E\left(\left[\rho ,P_{i}\right]\right)Q_{j}\right]| \end{array}$$

(A2) $$\begin{array}{cc} \; & =\frac{1}{2}\sum_{i,j}\left(\left|\mathrm{Tr}\left(\left\{\rho ,P_{i}\right\}-2P_{i}\rho P_{i}\right)E^{*}\left(Q_{j}\right)\right|+|\mathrm{Tr}\left[\rho ,P_{i}\right]E^{*}\left(Q_{j}\right)|\right), \end{array}$$

where Equation (A2) originates from the property of Hilbert–Schmidt adjoint $E^{*}$. Utilizing Equation (A2) and setting $\rho =\sum_{k}c_{k}\rho_{k}$, we proceed to derive

$$\begin{array}{cc} \; & M(P,Q;\sum_{k}c_{k}\rho_{k}) \end{array}$$

(A3) $$\begin{array}{cc} \; & =\frac{1}{2}\sum_{i,j}\left(\left|\mathrm{Tr}\left[\left\{\sum_{k}c_{k}\rho_{k},P_{i}\right\}-2P_{i}\left(\sum_{k}c_{k}\rho_{k}\right)P_{i}\right]E^{*}\left(Q_{j}\right)\right|+|\mathrm{Tr}\left[\sum_{k}c_{k}\rho_{k},P_{i}\right]E^{*}\left(Q_{j}\right)|\right) \end{array}$$

(A4) $$\begin{array}{cc} \; & ⩽\frac{1}{2}\sum_{i,j}\left(\sum_{k}c_{k}\left(\left|\mathrm{Tr}\left[\left\{\rho_{k},P_{i}\right\}-2P_{i}\rho_{k}P_{i}\right]E^{*}\left(Q_{j}\right)\right|+|\mathrm{Tr}\left[\rho_{k},P_{i}\right]E^{*}\left(Q_{j}\right)|\right)\right) \end{array}$$

(A5) $$\begin{array}{cc} \; & =\sum_{k}c_{k}\frac{1}{2}\sum_{i,j}\left(\left|\mathrm{Tr}\left(\left\{\rho_{k},P_{i}\right\}-2P_{i}\rho_{k}P_{i}\right)E^{*}\left(Q_{j}\right)\right|+|\mathrm{Tr}\left[\rho_{k},P_{i}\right]E^{*}\left(Q_{j}\right)|\right) \end{array}$$

(A6) $$\begin{array}{cc} \; & =\sum_{k}c_{k}M\left(P,Q;\rho_{k}\right), \end{array}$$

which implies the convexity of M(P,Q;ρ). Here, we apply the triangle inequality in Equation (A4). (iii) For any pair (i,j) such that $[P_{i},E^{*}\left(Q_{j}\right)]=0$, it follows from Equation (A2) that

(A7) $$\begin{array}{cc} M_{Re}\left(P,Q;\rho \right) & =\frac{1}{2}\sum_{i,j}\left|\mathrm{Tr}\left(\left\{\rho ,P_{i}\right\}-2P_{i}\rho P_{i}\right)E^{*}\left(Q_{j}\right)\right| \end{array}$$

(A8) $$\begin{array}{cc} \; & =\frac{1}{2}\sum_{i,j}\left|\mathrm{Tr}\left(\rho P_{i}+P_{i}\rho \right)E^{*}\left(Q_{j}\right)-2\mathrm{Tr}\left[P_{i}\rho P_{i}E^{*}\left(Q_{j}\right)\right]\right| \end{array}$$

(A9) $$\begin{array}{cc} \; & =\sum_{i,j}\left|\mathrm{Tr}\left[\rho P_{i}E^{*}\left(Q_{j}\right)\right]-\mathrm{Tr}\left[P_{i}\rho E^{*}\left(Q_{j}\right)P_{i}\right]\right| \end{array}$$

(A10) $$\begin{array}{cc} \; & =0. \end{array}$$

Meanwhile,

(A11) $$M_{Im}\left(P,Q;\rho \right)=\frac{1}{2}\sum_{i,j}|\mathrm{Tr}\left[\rho ,P_{i}\right]E^{*}\left(Q_{j}\right)|=0.$$

Similarly, if the condition $[P_{i},\rho ]=0$ holds for any index *i*, we can deduce that

(A12) $$\mathrm{Tr}\left[E\left(P_{i}\rho P_{i}\right)Q_{j}\right]=\mathrm{Tr}\left[E\left(\rho P_{i}^{2}\right)Q_{j}\right]=\mathrm{Tr}\left[E\left(\rho P_{i}\right)Q_{j}\right].$$

We thus have M(P,Q;ρ)=0 in both scenarios. Consequently, the condition M(P,Q;ρ)>0 signifies that there exists a pair (i,j) such that $P_{i}$ fails to commute with both ρ and $E^{*}\left(Q_{j}\right)$. (iv) We note that the condition $[D\left(\rho \right),P_{i}]=0$ leads to the conclusion M(P,Q;D(ρ))=0. Consequently, by the property (ii), we have

(A13) $$M(P,Q;D_{s}\left(\rho \right))⩽\left(1-s\right)\cdot M\left(P,Q;\rho \right)⩽M\left(P,Q;\rho \right)$$

for s∈[0,1]. □

## Appendix B. Proof of Theorem 1

The proof of Theorem 1 relies on the Lüders theorem; therefore, we will begin by revisiting this theorem.

**Theorem** **A1** ([43])**.** *Let H denote a complex separable Hilbert space, and let A be a self-adjoint operator possessing a discrete spectrum, characterized by the spectral decomposition $A=\sum_{i}a_{i}E_{i}$, where $a_{i}$ are distinct eigenvalues. The equality*

(A14) $$\mathrm{Tr}[I_{L}\left(\rho \right)\cdot B]=\mathrm{Tr}\left[\rho \cdot B\right]$$

*is satisfied for all states ρ if and only if the operator B commutes with every $E_{i}$, where B is any self-adjoint operator on H, and $I_{L}\left(\rho \right)$ denotes the Lüders transformation of the state ρ given by*

(A15) $$I_{L}\left(\rho \right)=\sum_{i}E_{i}\rho E_{i}.$$

**Proof of Theorem** **1.** It is evident that commutativity alone is adequate for establishing that M(P,Q;ρ)=0. Conversely, if M(P,Q;ρ)=0 is satisfied for every $\rho \in S(H_{A})$, then

(A16) $$\sum_{i=1}^{n}\mathrm{Tr}\left[E\left(\rho P_{i}\right)Q_{j}\right]=\sum_{i=1}^{n}\mathrm{Tr}\left[E\left(P_{i}\rho P_{i}\right)Q_{j}\right],$$

which indicates

(A17) $$\mathrm{Tr}[E^{*}\left(Q_{j}\right)\rho ]=\mathrm{Tr}\left[E^{*}\left(Q_{j}\right)\cdot \left(\sum_{i=1}^{n}P_{i}\rho P_{i}\right)\right].$$

Given that $0⩽E^{*}\left(Q_{j}\right)⩽1$, it follows that $E^{*}\left(Q_{j}\right)$ is a quantum effect for each *j*. According to Theorem A1, it follows that the commutation relation $[P_{i},E^{*}\left(Q_{j}\right)]=0$ is satisfied for every pair of indices (i,j). □

## Appendix C

**Proof of Proposition** **2.** We first note that there are equivalent expressions for $M_{Re}\left(P,Q;\rho \right)$ and $M_{Im}\left(P,Q;\rho \right)$ given by Equations (16) and (17), respectively. In fact, we have

$$\begin{array}{cc} \; & \left|\frac{1}{2}\mathrm{Tr}[E\left(\left\{\rho ,P_{i}\right\}\right)Q_{j}]-\mathrm{Tr}\left[E\left(P_{i}\rho P_{i}\right)Q_{j}\right]\right| \end{array}$$

(A18) $$\begin{array}{cc} \; & =\frac{1}{2}\left|\sum_{k}\left(\mathrm{Tr}[E\left(P_{k}\rho P_{i}\right)Q_{j}]+\mathrm{Tr}\left[E\left(P_{i}\rho P_{k}\right)Q_{j}\right]\right)-2\mathrm{Tr}[E\left(P_{i}\rho P_{i}\right)Q_{j}]\right| \end{array}$$

(A19) $$\begin{array}{cc} \; & =\frac{1}{2}\left|\sum_{k\neq i}\left(\mathrm{Tr}[E\left(P_{k}\rho P_{i}\right)Q_{j}]+\mathrm{Tr}[E\left(P_{i}\rho P_{k}\right)Q_{j}]\right)\right| \end{array}$$

(A20) =Re∑k≠iTr[E(PkρPi)Qj].

Thus, when summing over all indices i,j,k in Equation (A20), we have

(A21) MRe(P,Q;ρ)=∑jRe∑k≠iTr[E(PkρPi)Qj].

Similarly to $M_{Re}\left(P,Q;\rho \right)$, we also obtain

(A22) MIm(P,Q;ρ)=∑jIm∑k≠iTr[E(PkρPi)Qj].

It can be derived from Equations (A21) and (A22) that

(A23) M(P,Q;ρ)=∑jRe∑k≠iTr[E(PkρPi)Qj]+Im∑k≠iTr[E(PkρPi)Qj]

(A24) $$\begin{array}{cc} \; & ⩽\sqrt{2}\sum_{j}\sum_{k\neq i}\left|\mathrm{Tr}[E\left(P_{k}\rho P_{i}\right)Q_{j}]\right| \end{array}$$

(A25) $$\begin{array}{cc} \; & =\sqrt{2}\sum_{j}\sum_{k\neq i}\left|\langle p_{k}|\rho |p_{i}\rangle \langle p_{i}|E^{*}\left(Q_{j}\right)|p_{k}\rangle \right|, \end{array}$$

where Equation (A24) follows from the fact that |Re(z)|+|Im(z)|⩽2|z| for any z∈C and we apply the property of the Hilbert–Schmidt adjoint $E^{*}$ in Equation (A25). Employing the Cauchy–Schwarz inequality, we proceed to have

$$\begin{array}{cc} \; & \sum_{j}\sum_{k\neq i}\left|\langle p_{k}|\rho |p_{i}\rangle \langle p_{i}|E^{*}\left(Q_{j}\right)|p_{k}\rangle \right| \end{array}$$

(A26) $$\begin{array}{cc} \; & ⩽\sum_{j}\sqrt{\left(\sum_{k\neq i}{\left|\langle p_{k}|\rho |p_{i}\rangle \right|}^{2}\right)\cdot \left(\sum_{k\neq i}{\left|\langle p_{i}|E^{*}\left(Q_{j}\right)|p_{k}\rangle \right|}^{2}\right)} \end{array}$$

(A27) $$\begin{array}{cc} \; & =\sum_{j}\sqrt{\left(\sum_{k\neq i}{\left|\rho_{ki}\right|}^{2}\right)\cdot \left(\sum_{k\neq i}{\left|\langle p_{i}|E^{*}\left(Q_{j}\right)|p_{k}\rangle \right|}^{2}\right)}\;, \end{array}$$

where $\rho_{ki}:=\langle p_{k}|\rho |p_{i}\rangle$ is the matrix element of the state ρ. In the following, we will estimate the upper bound of

(A28) $$\sum_{k\neq i}{\left|p_{i}E^{*}\left(Q_{j}\right)p_{k}\right|}^{2}.$$

Equation (A28) can be reformulated as

(A29) $$\begin{array}{cc} \sum_{k\neq i}{\left|\langle p_{i}|E^{*}\left(Q_{j}\right)|p_{k}\rangle \right|}^{2} & =\sum_{k\neq i}\langle p_{i}|E^{*}\left(Q_{j}\right)|p_{k}\rangle \langle p_{k}|E^{*}\left(Q_{j}\right)|p_{i}\rangle \end{array}$$

(A30) $$\begin{array}{cc} \; & =\sum_{k\neq i}\mathrm{Tr}[P_{i}E^{*}\left(Q_{j}\right)P_{k}E^{*}\left(Q_{j}\right)] \end{array}$$

(A31) $$\begin{array}{cc} \; & =\mathrm{Tr}\left({\left[E^{*}\left(Q_{j}\right)\right]}^{2}\right)-\sum_{i=1}^{n}\mathrm{Tr}(P_{i}E^{*}\left(Q_{j}\right)P_{i}E^{*}\left(Q_{j}\right)) \end{array}$$

(A32) $$\begin{array}{cc} \; & =\mathrm{Tr}({\left[E^{*}\left(Q_{j}\right)\right]}^{2})-\sum_{i=1}^{n}{\left\langle p_{i}|E^{*}\left(Q_{j}\right)|p_{i}\right\rangle }^{2}. \end{array}$$

By applying the Jensen inequality, we obtain

(A33) $$\frac{\sum_{i=1}^{n}{\left\langle p_{i}|E^{*}\left(Q_{j}\right)|p_{i}\right\rangle }^{2}}{n}⩾{\left(\frac{\sum_{i=1}^{n}\langle p_{i}|E^{*}\left(Q_{j}\right)|p_{i}\rangle }{n}\right)}^{2}=\frac{1}{n^{2}},$$

where we note that $\sum_{i=1}^{n}\langle p_{i}|E^{*}\left(Q_{j}\right)|p_{i}\rangle =1$. It follows from Equations (A32) and (A33) that

(A34) $$\sum_{k\neq i}{\left|\langle p_{i}|E^{*}\left(Q_{j}\right)|p_{k}\rangle \right|}^{2}⩽1-\frac{1}{n}.$$

By integrating Equations (A25) and (A34), we ultimately derive

(A35) $$M\left(P,Q;\rho \right)⩽\sqrt{2}n\cdot \sqrt{\left(1-\frac{1}{n}\right)\cdot \left(\sum_{k\neq i}{\left|\rho_{ki}\right|}^{2}\right)}=\sqrt{2n\left(n-1\right)C_{l_{2}}\left(\rho \right)}\;,$$

where $C_{l_{2}}\left(\rho \right)=\sum_{k\neq i}{\left|\rho_{ki}\right|}^{2}$ is the $l_{2}$-norm coherence of ρ under the orthonormal basis ${\left\{|p_{i}\rangle \right\}}_{i=1}^{n}$ [39]. Finally, let us examine the conditions under which the upper bound can be achieved. The equality in Equation (A33) holds if and only if $\langle p_{i}|E^{*}\left(Q_{j}\right)|p_{i}\rangle =1/n$ for indices *i* and *j*. Furthermore, the condition for equality in Equation (A34) results in $\mathrm{Tr}({\left[E^{*}\left(Q_{j}\right)\right]}^{2})=1$, indicating that $E^{*}\left(Q_{j}\right)$ is a pure state. By combining these two findings, we can infer that $E^{*}\left(Q_{j}\right)$ is a maximally coherent state. The equality expressed in Equation (A26) is equivalent to the condition that $|\rho_{ki}|$ is proportional to $\left|\langle p_{i}|E^{*}\left(Q_{j}\right)|p_{k}\rangle \right|$ for all i,j,k∈{1,2,⋯,n}. Consequently, it can be concluded that $|\rho_{ki}|=1/n$ since $|\langle p_{i}|E^{*}\left(Q_{j}\right)|p_{k}\rangle |=1/n$. Hence, the upper bound is achieved if and only if $|\langle p_{i}|E^{*}\left(Q_{j}\right)|p_{k}\rangle |=|\rho_{ki}|=1/n$ for all i,j,k∈{1,2,⋯,n}. □

## Appendix D. Proofs of Theorems 2 and 3 and Corollary 1

Before proving the Theorem 2, it is necessary to introduce several auxiliary lemmas.

**Definition** **A1** ([55])**.** *Let A be a $C^{*}$-algebra; a linear map D on A is said to be a derivation if D(xy)=D(x)y+xD(y) holds for all x,y∈A.*

**Lemma** **A1** ([43])**.** *Let D:A→A be a bounded derivation on the unital $C^{*}$-algebra A. If $D^{\left(2\right)}\left(a\right)=D\left(D\left(a\right)\right)=0$ for some a∈A, then D(a) is quasi-nilpotent, i.e., the spectrum of D(a) is {0}.*

**Lemma** **A2.**
*Let H be a complex matrix, if [H,P]=0 holds for every projection P, where [·,·] is the commutator of two matrices. Then, H is a scalar matrix, i.e., H=γ·1 for some γ∈C.*

**Proof of Theorem** **2.** It has been shown that the spatiotemporal Born rule exists for the Lüders–von Neumann distribution $P_{i,j}\left(\rho_{A}\right)$ with respect to $\left(E,\rho_{A}\right)$ if and only if $P_{i,j}\left(\rho_{A}\right)=Q_{i,j}\left(\rho_{A}\right)$ for every TPSM scenario [26]. Furthermore, it can be noted that the condition $P_{i,j}\left(\rho_{A}\right)=Q_{i,j}\left(\rho_{A}\right)$ is equivalent to the statement $M_{Re}\left(P,Q;\rho_{A}\right)=0$. In the following, we will establish that if the channel E is injective and $M_{Re}\left(P,Q;\rho_{A}\right)=0$ for all TPSM schemes $\left\{\rho_{A},\left\{P_{i}\right\},E,\left\{Q_{j}\right\}\right\}$, then $\rho_{A}$ must be the completely mixed state 1/n. The condition $P_{i,j}\left(\rho_{A}\right)=Q_{i,j}\left(\rho_{A}\right)$ for all projective measurements $\left\{P_{i}\right\},\left\{Q_{j}\right\}$ is equivalent to the statement that

(A36) $$D_{i,j}\left(\rho_{A}\right)=P_{i,j}\left(\rho_{A}\right)-Q_{i,j}\left(\rho_{A}\right)=\frac{1}{2}\mathrm{Tr}[E\left(\rho_{A}-\rho_{i}\right)Q_{j}]=0$$

is satisfied for all projective measurements $\left\{P_{i}\right\},\left\{Q_{j}\right\}$, where

(A37) $$\rho_{i}=P_{i}\rho_{A}P_{i}+\left(1-P_{i}\right)\rho_{A}\left(1-P_{i}\right).$$

Consequently, one can conclude from the spectral theorem and the linearity of the trace that $E(\rho_{A}-\rho_{i})=0$ holds for all $\left\{P_{i}\right\}$. Given that the map E is injective, the condition $E(\rho_{A}-\rho_{i})=0$ necessitates that $\rho_{A}=\rho_{i}$ for each $\rho_{i}$, where $\rho_{i}$ is defined in Equation (A37). It is evident from Equation (A37) that

(A38) $$0=\rho_{A}-\rho_{i}=\rho_{A}P_{i}+P_{i}\rho_{A}-2P_{i}\rho_{A}P_{i}=\left[P_{i},\left[P_{i},\rho_{A}\right]\right]=D^{\left(2\right)}\left(\rho_{A}\right),$$

where the map D on A is defined as $D:T\mapsto [P_{i},T]$ for T∈A. It can be readily verified that D constitutes a bounded derivation on the $C^{*}$-algebra A. According to Equation (A38) and Lemma A1, it follows that $D(\rho_{A})=0$, which implies $[P_{i},\rho_{A}]=0$ for all projections $P_{i}$. Given that $\mathrm{Tr}(\rho_{A})=1$, we can conclude that $\rho_{A}=1/n$ by utilizing Lemma A2. □

**Proof of Theorem** **3.** It has been shown that if the bipartite state τ∈A⊗B is separable, then there exists a channel E such that $\tau =E★\rho_{A}$, where $\rho_{A}={\mathrm{Tr}}_{B}\left[\tau \right]$ denotes the reduced state of system *A* [48]. For such a channel E, the separability of τ implies that

(A39) $$\begin{array}{cc} \mathrm{Tr}[\tau \left(P_{i}⊗Q_{j}\right)] & =\mathrm{Tr}\left[\left(\sum_{k}t_{k}\rho_{A_{k}}⊗\rho_{B_{k}}\right)\left(P_{i}⊗Q_{j}\right)\right] \end{array}$$

(A40) $$\begin{array}{cc} \; & =\sum_{k}t_{k}\mathrm{Tr}[\left(\rho_{A_{k}}P_{i}\right)⊗\left(\rho_{B_{k}}Q_{j}\right)] \end{array}$$

(A41) $$\begin{array}{cc} \; & =\sum_{k}t_{k}\mathrm{Tr}(\rho_{A_{k}}P_{i})\cdot \mathrm{Tr}(\rho_{B_{k}}Q_{j}) \end{array}$$

(A42) $$\begin{array}{cc} \; & ⩾0, \end{array}$$

where $\tau =\sum_{k}t_{k}\rho_{A_{k}}⊗\rho_{B_{k}}$, $\sum_{k}t_{k}=1$, $t_{k}⩾0$ and $\rho_{A_{k}}\in A,\rho_{B_{k}}\in B$. In Equation (A41), we utilize the relation Tr(X⊗Y)=Tr(X)·Tr(Y), where X∈A and Y∈B. On the other hand, it is straightforward to calculate that

(A43) $$\begin{array}{cc} \mathrm{Tr}[\tau \left(P_{i}⊗Q_{j}\right)] & =\mathrm{Tr}[E★\rho_{A}\left(P_{i}⊗Q_{j}\right)] \end{array}$$

(A44) $$\begin{array}{cc} \; & =\frac{1}{2}\mathrm{Tr}[\left\{\rho_{A}⊗1_{B},J\left[E\right]\right\}\left(P_{i}⊗Q_{j}\right)] \end{array}$$

(A45) $$\begin{array}{cc} \; & =\frac{1}{2}\mathrm{Tr}[J\left[E\right]\left(\left\{\rho_{A},P_{i}\right\}⊗Q_{j}\right)] \end{array}$$

(A46) $$\begin{array}{cc} \; & =\frac{1}{2}\mathrm{Tr}[E\left(\left\{\rho_{A},P_{i}\right\}\right)Q_{j}] \end{array}$$

(A47) =ReTr[E(ρAPi)Qj],

where Equation (A45) follows from the fact that

(A48) Tr[J[E](X⊗Y)]=Tr[E(X)Y],X∈A,Y∈B.

Consequently, under the given TPSM scenario $\left\{\rho_{A},\left\{P_{i}\right\},E,\left\{Q_{j}\right\}\right\}$, it follows that ReTr[E(ρAPi)Qj]⩾0. □

**Proof of Corollary** **1.** It has been shown that there exists a unique element $\varrho_{AB}$, which conforms to the expression in Equation (43), such that two-time expectation value is given by

(A49) $${\langle P,Q\rangle }_{\left(E,\rho_{A}\right)}=\mathrm{Tr}[\varrho_{AB}\left(P⊗Q\right)]$$

for the light-touch observable P∈A and any observable Q∈B [22]. We then derive

(A50) ReTr[E(ρAP)Q]=〈P,Q〉(E,ρA)=∑ipiTr[E(PiρAPi)Q]=〈P,Q〉,

where the first equality is elaborated in Equation (A47) and the last equality is due to the temporal compatibility. □
